# Frequency of five *Escherichia Coli* pathotypes in Iranian adults and children with acute diarrhea

**DOI:** 10.1371/journal.pone.0245470

**Published:** 2021-02-04

**Authors:** Sana Eybpoosh, Saeid Mostaan, Mohammad Mehdi Gouya, Hossein Masoumi-Asl, Parviz Owlia, Babak Eshrati, Mohammad Reza Montazer Razavi Khorasan, Saeid Bouzari

**Affiliations:** 1 Department of Epidemiology and Biostatistics, Research Centre for Emerging and Reemerging Infectious Diseases, Pasteur Institute of Iran, Tehran, Iran; 2 Department of Molecular Biology, Pasteur Institute of Iran, Tehran, Iran; 3 Centre for Communicable Diseases Control, Ministry of Health and Medical Education, Tehran, Iran; 4 Molecular Microbiology Research Center, Shahed University, Tehran, Iran; 5 Center for Preventive Medicine, Department of Social Medicine, Iran University of Medical Sciences, Tehran, Iran; Public Health England, UNITED KINGDOM

## Abstract

**Background:**

Knowledge about the distribution of *Escherichia Coli* (*E*. *coli*) pathotypes in Iran is limited. This nation-wide survey aims to provide a comprehensive description of the distribution of five pathogenic *E*. *coli* in Iran.

**Methods:**

Stool samples were collected from 1,306 acute diarrhea cases from 15 provinces (2013–2014). *E*. *coli*-positive cultures underwent PCR testing for the detection of STEC, ETEC, EPEC, EAEC, and EIEC pathotypes. Pathotype frequency by province, age-group, and season was estimated.

**Results:**

979 diarrhea samples (75.0%) were culture-positive for *E*. *coli* (95% CI: 72.6, 77.3%), and 659 (50.5%) were pathogenic *E*. *coli* (95% CI: 47.8, 53.2%). STEC was the most frequent pathotype (35.4%). ETEC (14.0%) and EPEC (13.1%) were the second and the third most frequent pathotypes, respectively. EAEC (4.3%) and EIEC (0.3%) were not highly prevalent. Fars (88.7%) and Khorasan-e-Razavi (34.8%) provinces had the highest and lowest frequencies, respectively. *E*. *coli* pathotypes were more frequent in warmer than cooler seasons, showed the highest frequency among children under five years of age (73%), and had no significant association with participants’ gender.

**Conclusions:**

Diarrheagenic *E*. *coli* may be an important cause of acute diarrhea in adults and children in Iran. STEC and ETEC seem to be widespread in the country with a peak in warmer seasons, impacting the recommended use of seasonal STEC and ETEC vaccines, especially in high-risk groups. Monitoring the incidence of *E*. *coli* pathotypes, serotypes, and antibiotic resistance over time is highly recommended for evaluation of interventions.

## Introduction

A subset of *Escherichia coli* (*E*. *coli*) bacteria, called diarrheagenic *E*. *Coli* (DEC), is capable of causing diarrhea in the human host. Different DEC strains (pathotypes) have been determined recently, some of which include Shiga toxin–producing *E*. *coli* (STEC), enteropathogenic *E*. *coli* (EPEC), enterohemorrhagic *E*. *coli* (EHEC), *enterotoxigenic E*. *coli* (ETEC), enteroaggregative *E*. *coli* (EAEC), enteroinvasive *E*. *coli* (EIEC), and diffusely adherent *E*. *coli* (DAEC) [1].

Current evidence suggests that the incidence of *E*. *coli* pathotypes may vary by geographic region. Some of the STEC serotypes, called O157:H7, are prevalent in the United States and Canada. The non-O157:H7 serotypes, however, are prevalent in Latin America and Europe. ETEC, is a widespread pathotype in the developing world [2].

In Iran, there is a paucity of large-scale studies providing an unbiased estimate of the distribution of major *E*. *Coli* pathotypes over the country [3]. Many of the available studies have focused on the infection of infants or children under five years of age [4–9], but only few studies are available from adolescents and adults. Studies conducted in Iran also have some methodological limitations, such as small sample sizes, limited sampling locations, and/or investigation of few *E*. *Coli* pathotypes [10–13]. Also, the sampling method of most available studies is non-probabilistic. All the above issues can adversely affect the generalizability of these studies’ results. In this study, we conducted a nationally-representative survey on all age groups in order to illuminate the role of five *E*. *Coli* pathotypes in the epidemiology of diarrheal diseases in different geographical regions and among adults, adolescents, and children of Iran.

## Material and method

### Ethics statement

This study was approved by the ethics committee of Pasteur Institute of Iran (Ethical code: IR.PII.REC.1394.85). Participants were informed about the study objectives, assured about the confidentiality of their information, and gave their written informed consent for participation in this study.

### Study design

Cross-sectional.

### Study population

Consisted of all Iranian nationals who were residents of target provinces of Iran and referred to health centers with a chief complaint of acute diarrhea (with/without bleeding). Acute diarrhea was defined as passage of liquid/watery stool for more than 2–3 times a day. Immunologically compromised patients as well as patients with chronic diarrhea were excluded. Also cases in which individuals traveled two weeks prior to the process of recruitment were not included.

### Sampling procedure

Of 31 provinces in Iran, 15 were selected in this study (sampling fraction = 50%). The provinces were selected in a way that ensured that all the geographical areas of Iran are included. In each province, three cities were selected, leading to a total number of 45 cities. Stool samples from diarrhea cases were collected at the second half of each month in order to harmonize the time-frame of sampling across all cities. A random sample of collected specimens was then subjected to culture. *E*. *coli*-positive samples identified by culture method were sent to the National *E*. *Coli* Reference Laboratory (NECRL) in Pasteur Institute of Iran (PII) for molecular analysis and pathotype identification.

To ensure data quality and standardize the laboratory procedures across all field centers, we held a series of training workshops where the staff were thoroughly trained about the study procedures. Trainings involved how to collect stool samples, do the cultures, interpret and record culture results, and transfer the samples to NECRL.

### Isolation and Identification of *E*. *coli*

#### Culture

Stool samples were inoculated on MacConkey agar (Merck, Catalog No. 105465) at 37 °C for 24 hours. After O/N incubation (incubator: Vision Korea), results were checked, and five typical colonies on MacConkey agar (with pink color) were selected and transferred to the triple sugar iron agar media (Merck, Catalog No. 104728). Colonies with *E*. *coli* characteristics were selected and transferred to SIM medium (Merck, Catalog No. 105470), Simmons citrate (Merck, Catalog No.102501), and MRVP broth (Merck, Catalog No. 105712) to further check for the presence of *E*. *coli* characteristics. Samples that were Indol-positive (Kovacs, Merck, Catalog No. 109293), MR-positive (Methyl red, Merck, Catalog No. 106076), VP-negative (KOH, SIGMA USA, Catalog No. P5958 and alpha naphthol-1 Catalog No. 70480), and citrate-negative were potentially considered *E*. *coli*-positive. Identified *E*. *coli-*positive samples were incubated on LB (Merck, Catalog No. 110285). After O/N incubation, the samples were centrifuged (Eppendorf Germany) and the final products were stored at −70 °C until used for PCR assays.

#### PCR

Eight *E*. *coli* virulence genes indicative of the five candidate *E*. *coli* pathotypes were subjected to PCR assay. The primers used for probe amplification were chosen either from existing literature or designed from available gene sequences (Table 1).

**Table 1:** Target genes and their characteristics for isolation of different pathotypes.

Pathotype	Target Gene(s)	Amplicon Size (bp)	Primers	
EPEC	*Eae*	544	Primer (Forward)	CTGAACGGCGATTACGCGAA
			Primer (Reverse)	CGAGACGATACGATCCAG
	*Bfp*	910	Primer (Forward)	GACACCTCATTGCTGAAGTCG
			Primer (Reverse)	CCAGAACACCTCCGTTATGC
ETEC	*Lt*	655	Primer (Forward)	GAACAGGAGGTTTCTGCGTTAGGTG
			Primer (Reverse)	CTTTCAATGGCTTTTTTTTGGGAGTC
	*St*	157	Primer (Forward)	CCTCTTTTAGCCAGACAGCTGAATCACTTG
			Primer (Reverse)	CAGGCAGGATTACAACAAAGTTCACAG
STEC	*Eae*	544	Primer (Forward)	CAGGCAGGATTACAACAAAGTTCACAG
			Primer (Reverse)	CAGGCAGGATTACAACAAAGTTCACAG
	*Stx1*	244	Primer (Forward)	CGATGTTACGGTTTGTTACTGTGACAGC
			Primer (Reverse)	AATGCCACGCTTCCCAGAATTG
	*Stx2*	324	Primer (Forward)	GTTTTGACCATCTTCGTCTGATTATTGAG
			Primer (Reverse)	AGCGTAAGGCTTCTGCTGTGAC
EAEC	*AA*	629	Primer (Forward)	CTGGCGAAAGACTGTATCAT
			Primer (Reverse)	CAATGTATAGAAATCCGCTGTT
EIEC	*invE*	766	Primer (Forward)	CGATAGATGGCGAGAAATTATATCCCG
			Primer (Reverse)	CGATCAAGAATCCCTAACAGAAGAATCAC

For the preparation of samples, ten *μl* Master Mix 2X (Fermentas, Catalog No. K0171) plus seven *μl* of DDW and forward and reverse primers (1 *μl* of each) were added to one *μl* of the sample. For positive and negative controls, 100 kb DNA ladder and ladder mix were used. The processes of denaturation, annealing and extension were performed in the Eppendorf thermo cycler (Germany).

### Statistical analysis

Data were analyzed in Stata software (version 14). Frequency of *E*. *coli* pathotypes was estimated in the overall population and in subgroups of age, location (province/city), and time (4 seasons and 12 months). Association of each *E*. *coli* pathotype with season and type of diarrhea (with/without bleeding) was assessed using Chi Square test. Statistical tests were considered as significant at 0.05 levels.

## Results

Between January 2013 and January 2014, 1,305 diarrheal samples were collected from 15 provinces of Iran. Of these, 979 samples were *E*. *coli*-positive (75.0%; 95% CI: 72.6, 77.3%), and were subjected to molecular assays. Pathogenic *E*. *coli* were detected in 659 out of 1,305 diarrheal samples (50.5%; 95% CI: 47.8, 53.2%). *Stx1* (26.1%) and *Eae (*25.9%) were the most frequent virulence genes. STEC (35.4%) was the most and EIEC (0.3%) was the least frequent pathotype (Table 2).

**Table 2:** Prevalence of five *E*. *coli* pathotypes and their virulence genes in Iran.

		Prevalence in diarrheal samples*	Frequency in *E*. *coli*-positive samples**
Pathotype	n	% (95% CI)	% (95% CI)
**STEC**	347	26.6 (24.2, 29.1)	35.4 (32.4, 38.5)
**ETEC**	137	10.5 (8.9, 12.3)	14.0 (11.9, 16.3)
**EPEC**	129	9.9 (8.3, 11.6)	13.2 (11.1, 15.5)
**EAEC**	43	3.3 (2.4, 4.4)	4.4 (3.2, 5.9)
**EIEC**	3	0.2 (0.1, 0.7)	0.3 (0.001, 0.9)
**Total**	659	50.5 (47.7, 53.2)	67.3 (64.3, 70.3)
**Virulence genes**			
***Stx1***	255	19.5 (17.4, 21.8)	26.1 (23.3, 28.9)
***Eae***	254	19.5 (17.3, 21.7)	25.9 (23.2, 28.8)
***Stx2***	201	15.4 (13.5, 17.5)	20.5 (18.0, 23.2)
***LT***	80	6.1 (4.9, 07.6)	8.2 (6.5, 10.1)
***ST***	78	6.0 (4.8, 7.4)	8.0 (6.5, 9.8)
***AA***	43	3.3 (2.4, 4.4)	4.4 (3.2, 5.9)
***Bfp***	5	0.4 (0.1, 0.9)	0.5 (0.2, 1.2)
***invE***	4	0.3 (0.1, 0.8)	0.4 (0.1, 1.0)

* Frequency is calculated by dividing the numbers to the total number of diarrheal cases sampled (n = 1305).

** Frequency is calculated by dividing the numbers to the total number of *E*. *coli*-positive samples identified in culture (n = 979).

### Geographical distribution of pathogenic *E*. *coli* in Iran

**Fig 1:**
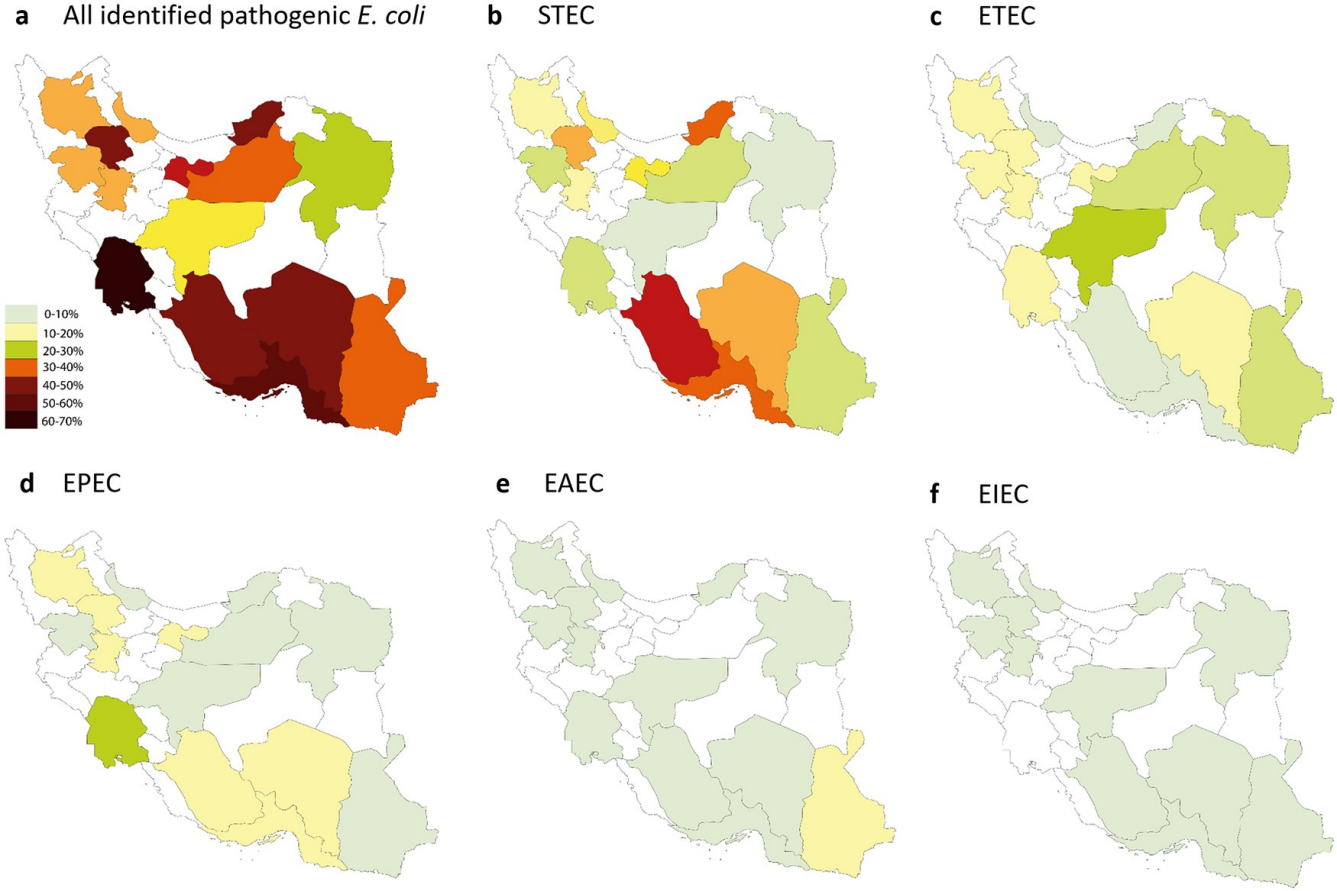
Frequency of *E*. *coli* pathotypes in 979 *E*. *coli*-positive culture samples collected from 15 provinces of Iran.

Pathogenic *E*. *coli* were detected in all investigated provinces. The overall frequency of *E*. *coli* pathotypes was highest in Fars province (88.7%), and lowest in Razavi Khorasan Province, although the prevalence in the latter province was still considerably high (34.8%; Fig 1, Panel A, and S1 File). At the city level, 100% of the received samples from Gorgan, Tarem (each with 3 received samples), and Abadeh (with 9 received samples) were pathogenic *E*. *coli*. Pathogenic *E*. *coli* were not detected in three cities, including Najaf-Abad, Saravan, and Damghan (with 5, 2, and 1 received samples, respectively; S2 File).

#### STEC

Was the most frequent pathotype (35.4%) in Iran and was detected in all provinces (Fig 1, Panel B). The highest and lowest frequency of STEC was observed in Fars (72.5%) and Esfahan (5.5%) provinces, respectively (Fig 1, Panel B and S1 File). At the city-level, STEC was detected in 33 cities (76.7%; Fig 1, Panel A, and S2 File).

#### ETEC

Was the second most frequent pathotype (14.0%) in Iran and was detected in all investigated provinces (Fig 1, Panel C). Esfahan (31.4%) and Fars (1.6%) provinces had the highest and lowest frequencies of this pathotype (Fig 1, Panel C and S1 File). ETEC was detected in twenty-nine cities (67.4%), and was the predominant pathotype in nine of them (Fig 1, Panel B, and S2 File).

#### EPEC

Was the third most prevalent *E*. *coli* pathotype in Iran (13.1%). The highest frequency (31.0%) was observed in Khuzestan province. This pathotype was not detected in Esfahan province (Fig 1, Panel D and S1 File). At the city-level, EPEC was observed in twenty-eight cities (65.1%), and was the predominant pathotype in four of them (Fig 1, Panel C, and S2 File).

#### EAEC

Was not highly prevalent in Iran (overall frequency = 4.3%). Except Kurdistan province with sixteen isolated EAEC pathotypes, other provinces identified less than five EAEC isolates (Fig 1, Panel E, and S1 File). At the city level, EAEC was detected in 19 out of 45 cities (44.2%), most of which had one EAEC isolate (Fig 1, Panel D, and S2 File).

#### EIEC

Was a rare pathotype in Iran (overall frequency = 0.3%; Fig 1, Panel F). The three positive strains identified in our sample belonged to Sirjan (0.9%), Andimeshk (0.6%), and Semnan (0.2%) cities (Fig 1, Panel E, and S2 File).

### Temporal variation in the prevalence of *E*. *coli* pathotypes

**Fig 2:**
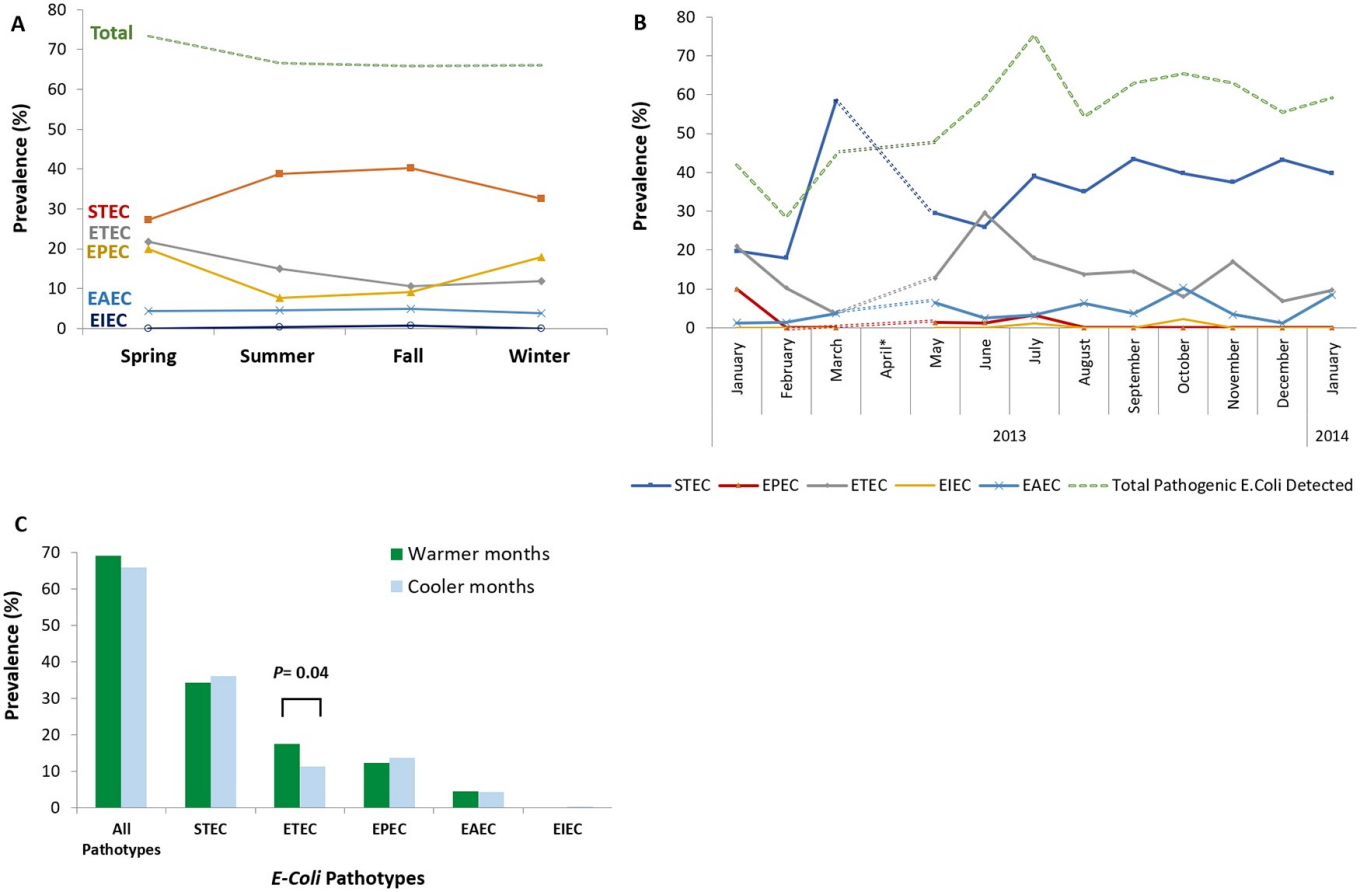
Seasonal trend of *E*. *coli* pathotypes in Iran. (a) Frequency of five *E*. *coli* pathotypes in four seasons. STEC was more prevalent during summer and fall, and ETEC usually showed a peak during spring and summer. Remarkable seasonal trends could not be observed for other pathotypes, probably due to scarcity of available data. (B) Frequency of five *E*. *coli* pathotypes in different months of a year. STEC showed a peak in March and ETEC showed a peak in June. Remarkable monthly trends could not be observed for other pathotypes, probably due to availability of very few samples. (C) Difference in the frequency of five *E*. *coli* pathotypes in warmer (spring and summer) versus cooler seasons (fall and winter). In overall, *E*. *coli* pathotypes were more frequently observed in warmer than cooler seasons but the difference was not statistically significant (*P* value = 0.321). This pattern was observed for the ETEC and EAEC pathotypes and was statistically significant for ETEC (*P* value = 0.04). STEC and EPEC were more frequently observed in cooler than warmer seasons but the difference, was negligible and not statistically significant.

STEC was more prevalent during summer and fall, with a peak in March, and ETEC was usually prevalent during spring and summer, with a peak in June. The seasonal pattern observed for the ETEC was statistically significant (17.6 vs. 11.3%, *P* value = 0.047). No remarkable seasonal and monthly trends could be identified for other pathotypes, probably due to availability of few samples (Fig 2; S3 File).

### Age-specific prevalence of *E*. *coli* pathotypes

The highest frequency of pathogenic *E*. *coli* was observed in infants and children under five years of age (73% each) with STEC (40.5% and 41.1%, respectively) and EIEC (2.7% and 0.4%, respectively) being the most frequent pathotypes. EPEC was highly frequent in children aged 1–5 years, as well as adults aged 30–40 years (16.7% each). ETEC had the highest frequency in children aged 5–10 years (21.4%). This pathotype was also common in adults and geriatrics (see Table 3).

**Table 3:** Age-specific frequency of *E*. *coli* pathotypes in 979 *E*. *coli*-positive culture samples received from 15 provinces of Iran.

Age group	All pathotypes	STEC	EPEC	ETEC	EIEC	EAEC	Received samples
Year	n (%)	n (%)	n (%)	n (%)	n (%)	n (%)	N
**0–1**	27 (73.0)	15 (40.5)	4 (10.8)	4 (10.8)	1 (2.7)	0 (0.0)	40
**1–5**	197 (73.0)	111 (41.1)	45 (16.7)	28 (10.4)	1 (0.4)	12 (4.4)	273
**5–10**	45 (64.3)	19 (27.1)	8 (11.4)	15 (21.4)	0 (0.0)	3 (4.3)	70
**10–20**	86 (71.1)	39 (32.2)	16 (13.2)	23 (19.0)	0 (0.0)	8 (6.6)	121
**20–30**	88 (67.2)	47 (35.9)	18 (13.7)	18 (13.7)	0 (0.0)	5 (3.8)	131
**30–40**	65 (67.7)	31 (32.3)	16 (16.7)	13 (13.5)	0 (0.0)	5 (5.2)	96
**40–50**	46 (54.1)	27 (31.8)	7 (8.2)	9 (10.6)	1 (1.2)	2 (2.4)	85
**50–60**	33 (64.7)	19 (37.3)	3 (5.9)	10 (19.6)	0 (0.0)	1 (2.0)	51
**60–70**	32 (66.7)	15 (31.3)	4 (8.3)	9 (18.8)	0 (0.0)	4 (8.3)	48
**> 70**	40 (57.1)	21 (30.0)	8 (11.4)	8 (11.4)	0 (0.0)	3 (4.3)	70
**Total**	659 (67.3)	347 (35.1)	129 (13.2)	137 (14.0)	3 (0.3)	43 (4.4)	979

## Discussion

In this nationally-representative survey, we estimated that 75% of acute diarrhea cases were culture-positive for *E*. *coli*, and 50.5% were infected with a pathogenic *E*. *coli* species. The study has yielded several findings about the frequency and spatio-temporal variation of *E*. *coli* pathotypes among diarrhea cases in Iran. Given this study focused on five pathotypes, the negative samples may still be infected with other *E*. *Coli* pathotypes that were not investigated in this study.

STEC was the predominant pathotype in our sample, with a high prevalence in infants and children under five years of age. In terms of time, STEC is one of the first identified *E*. *coli* pathotypes in Iran. The first report in this regard is published in 1998, where a prevalence of 4.9% was reported in the general population of western Iran [14]. A few years later, STEC prevalence was estimated at 0.7% in the general population of Golestan and Mazandaran provinces (North of Iran) [15]. Both studies used cytotoxic methods for identification of STEC pathotypes in the Vero cells.

We observed considerable heterogeneity in STEC frequency among investigated provinces (see Figs 1 and 2). This is consistent with the previous studies conducted in Iran by Aslani, et al. (1998 and 2003). Geographical heterogeneity in the distribution of STEC have been reported in other countries, as well [16, 17]. It is well acknowledged that STEC is mostly common in children under five years of age. Therefore, the heterogeneity of STEC prevalence across 15 provinces could be attributed to heterogeneous age distribution of patients sampled in this study. In addition, differences in STEC prevalence in the studied provinces might be attributed to the heterogeneity in reservoir infection across the country, as observed for infectious diseases as well [18]. Cattle and other ruminants are the natural reservoirs of STEC strains [19]. Transmission to human occurs through direct or indirect contact with the animal feces or through ingestion of contaminated food or water [20–22]. These factors would not be homogenous across provinces of Iran, and may justify the heterogeneity in STEC prevalence across the country. Further studies are required to investigate the extent of reservoir infection and environmental contamination in Iran.

Frequency of the STEC pathotype was estimated at 7.8% in diarrheal children under five years of age. This is lower than previous estimates in Iran [23–27], but is consistent with the estimates in China [28, 29] and Romania [24, 30]. Molecular tests were used as the diagnostic method in all of these studies. These results further highlight the role of factors other than age in STEC infection in Iran. In a study in Brazil that hierarchically analyzed data from 3,725 children under five years of age, authors highlighted the association of environmental factors, number of people per room, maternal age, and the age of the child with diarrhea [31]. A similar study in Kenya (2013) highlighted the role of hand hygiene of the child’s care-giver(s), drinking untreated water from the river, and lack of exclusive breastfeeding as predictors of childhood diarrhea [32].

ETEC is more prevalent in low- and middle-income countries and is a major pathogenic strain in travelers’ diarrhea, with a high burden in children of these countries [33]. In our study, ETEC showed a high prevalence in our total sample (the second most prevalent pathotype) as well as in the children less than five years of age. This is in agreement with the trend in the middle income countries [3, 34]. In a recent systematic review in Iran, the ETEC is identified as one of the most prevalent pathotypes in different parts of the country with an estimated pooled prevalence of 16% (95% CI: 11%–23%) [35]. Previous studies in Iran also reported ETEC as a frequent cause of diarrhea in children younger than five years of age [9, 36–39].

ETEC prevalence in many developing countries seems to be similar to the situation of Iran. Studies show that the incidence of diarrhea due to ETEC is high in children below one year of age, remains high among children between one and five years, and declines thereafter. Our findings are also supported by a systematic review of ETEC prevalence in the developing countries that reported ETEC at a constant high rate in children below five years of age [9, 37–40].

EPEC was the third most frequent pathotype in our study. It was also highly frequent in children under five years of age. EPEC is known to be a major cause of diarrhea in children, especially those below two years of age, with a prevalence of about 5–10% [41]. Prevalence of EPEC in Iranian children has been estimated by few studies. Two studies in this regard has reported an EPEC prevalence of 7% in this age group [42, 43]. Two other studies on diarrheal children attending hospitals reported an EPEC prevalence of 12.6% and 23% in this age group [24, 25]. Higher prevalence of EPEC reported in the two latter studies, which selected hospitalized diarrheal children, might be indicative of the role of EPEC pathotype in severe diarrhea. Prevalence of EPEC in Iranian adults was estimated at 9.9% in our study which is consistent with the estimate of a relevant systematic review in this field (11%; 95% CI: 8%–14%) [35].

In our sample, EAEC was not frequently observed, either in adults or in children. EAEC also constituted a small proportion of DEC pathotypes (about 6%). This is lower than the previous estimates in different parts of Iran, such as Tehran (20%) [44] Zanjan (25.6%) [11], and Tabriz (28.3%) [45]. It should be acknowledged that EAEC has recently been identified as a DEC pathotype that basically plays a major role in travelers’ diarrhea [40]. Given we have not included travelers; the lower EAEC frequency observed in our study may be attributed to our inclusion criteria. A study in Northern provinces of Iran that similarly excluded travelers from their samples, observed no cases with EAEC among diarrheal patients who referred to community outpatient clinics [46]. Some of the studies that have reported high prevalence of EAEC are based on hospitalized cases with diarrhea (e.g., [25]). This may suggest that EAEC infection is associated with more severe forms of diarrhea, leading to hospital admission. However, the data in this regard is limited and controversial. So, the role of EPEC and EAEC in severe diarrhea, especially among young children, remains an open area for future research.

We observed a prevalence of 0.3% for the EIEC pathotype in our study. Two out of three identified EIEC isolates identified in our study were detected in children less than five years of age. This is in agreement with previous estimates in children of this age group in Iran (Shiraz: 2.5% [47]; Sanandaj: 4.6% [48]. Human is the sole reservoir for the EIEC pathotype. Its transmission also requires high load of the pathogen, which decreases the chance of human-to-human transmission. Given these features and the very low prevalence of the pathotype in our study, which possesses a representative sample from Iran, it seems that infection with EIEC is a less important issue in Iran.

Our results also showed that the five *E*. *coli* pathotypes investigated in our study may be more prevalent in warmer than cooler seasons in Iran. This is in agreement with previous studies in the country [3, 9–11]. Seasonal variation in the incidence of pathogenic *E*. *Coli* has also been reported in Mexico [49], Kenya [50], and Taiwan [51], with the pathotypes being more frequent in the dry seasons than in the wet seasons. A global meta-analysis on 28 individual studies reported 8% increase in the incidence of diarrheagenic *E*. *coli* per 1°^C^ increase in the mean monthly temperature. After controlling for the effect of temperature, there was no significant association between rainfall and diarrheagenic *E*. *coli* incidence [52]. Iran has a hot, dry climate characterized by long, hot, dry summers and short, cool winters. Therefore, higher prevalence of *E*. *coli* pathotypes observed in warmer seasons in Iran may be explained by the high temperature and dryness of the weather in warmer seasons. These epidemiological findings could impact the recommended use of *E*. *coli* vaccines during warmer months. However, additional studies using pathotype-specific vaccines would be needed to further illuminate the possible benefits during lower acquisition rate seasons. The difference between ETEC-EAEC and STEC-EPEC rates in terms of seasonality suggests that the two pathotype groups may have different pathways of transmission and reservoirs in Iran.

This study has notable strengths. Most studies on this topic in Iran are limited to one or two DEC pathotypes, in a specific age group and limited geographical areas. To the best of our knowledge, this is the first study that provides a nationally-representative sample of diarrhea cases, which also includes all age groups and five major DEC pathotypes. Half of the provinces in Iran are included in this study. Our sampling also covered a time interval of 12 months, providing the opportunity to assess seasonality.

This study has also a number of limitations. Due to financial constraints and large-scale nature of the study, we did not identify the serotype of isolated *E*. *coli*. Hence, the results represent the frequency estimates that are based on molecular detection of *E*. *coli* pathotypes. Further studies on the O, K, and H antigens would provide a clearer picture of the distribution of major *E*. *coli* serotypes in Iran. Also, antibiotic resistance profile of isolated *E*. *coli* was not investigated in this study. Finally, the sampling method as well as the pathogen isolation technique are heterogenous across available studies, which limits our ability to compare the results of the studies. So, we could not derive conclusions about prevalence trends. This highlights the need for development of similar national-level evaluations in the future.

## Conclusion

Our results suggest that diarrheagenic *E*. *coli* may be an important cause of acute diarrhea both in adults and children in Iran. STEC and ETEC seem to be widespread in Iran, with a peak in warmer seasons. This epidemiological finding could impact the recommended use of STEC and ETEC vaccines during warmer seasons, especially for infants, young children and the elderly. EPEC and EAEC seem to be less prevalent, and EIEC seems to be a rare pathotype among Iranian outpatients with acute diarrhea. Continued national surveys are recommended for evaluations of time-trends and effectiveness of interventions.

## Supporting information

S1 File Frequency of *E. coli* pathotypes in 15 selected provinces of Iran. (DOCX)

S2 File Frequency of *E. coli* pathotypes in 43 selected cities of Iran. (DOCX)

S3 File Seasonal trend of *E. coli* pathotypes in 15 selected provinces of Iran. (DOCX)

## References

[1] Kaper J, Nataro J, Mobley H. Pathogenic Escherichia coli. Nature reviews Microbiology. 2004;2(2):123 10.1038/nrmicro818 15040260

[2] Keskimäki M, Mattila L, Peltola H, Siitonen A. Prevalence of diarrheagenic Escherichia coli in Finns with or without diarrhea during a round-the-world trip. Journal of clinical microbiology. 2000;38(12):4425–9. 10.1128/JCM.38.12.4425-4429.2000 11101575 PMC87616

[3] Jafari A, Aslani M, Bouzari S. Escherichia coli: a brief review of diarrheagenic pathotypes and their role in diarrheal diseases in Iran. Iranian journal of microbiology. 2012;4(3):102 23066484 PMC3465535

[4] Kalantar E, SOHEYLI F, Salimi H, SOLTAN DMM. Frequency, antimicrobial susceptibility and plasmid profiles of Escherichia coli pathotypes obtained from children with acute diarrhea. 2011.

[5] Pourakbari B, Heydari H, Mahmoudi S, Sabouni F, Teymuri M, Ferdosian F, et al Diarrhoeagenic E. coli pathotypes in children with and without diarrhoea in an Iranian referral paediatrics centre. 2013. 24975306

[6] Khoshvaght H, Haghi F, Zeighami H. Extended spectrum betalactamase producing Enteroaggregative Escherichia coli from young children in Iran. Gastroenterology and Hepatology from bed to bench. 2014;7(2):131 24834305 PMC4017568

[7] Mitra M, Mehdi R, Hosein A, Ahmad K. Multiple drug resistance of enteropathogenic Escherichia coli isolated from children with diarrhea in Kashan, Iran. African Journal of Microbiology Research. 2011;5(20):3305–9.

[8] Heidary M, Momtaz H, Madani M. Characterization of diarrheagenic antimicrobial resistant Escherichia coli isolated from pediatric patients in Tehran, Iran. Iranian Red Crescent Medical Journal. 2014;16(4). 10.5812/ircmj.12329 24910786 PMC4028759

[9] Alizade H, Ghanbarpour R, Aflatoonian MR. Molecular study on diarrheagenic Escherichia coli pathotypes isolated from under 5 years old children in southeast of Iran. Asian Pacific Journal of Tropical Disease. 2014;4:S813–S7.

[10] Alikhani MY, Hashemi SH, Aslani MM, Farajnia S. Prevalence and antibiotic resistance patterns of diarrheagenic Escherichia coli isolated from adolescents and adults in Hamedan, Western Iran. Iranian journal of microbiology. 2013;5(1):42 23466523 PMC3577554

[11] Bafandeh S, Haghi F, Zeighami H. Prevalence and virulence characteristics of enteroaggregative Escherichia coli in a case–control study among patients from Iran. Journal of medical microbiology. 2015;64(5):519–24. 25813820 10.1099/jmm.0.000055

[12] Alizade H, Sharifi H, Naderi Z, Ghanbarpour R, Bamorovat M, Aflatoonian MR. High frequency of diarrheagenic Escherichia coli in HIV-infected patients and patients with thalassemia in Kerman, Iran. Journal of the International Association of Providers of AIDS Care (JIAPAC). 2017;16(4):353–8. 10.1177/2325957415617831 26590202

[13] Alizadeh A, Behrouz N, Salmanzadeh S, Ranjbar M, Azimian M, Habibi E, et al Escherichia coli, Shigella and Salmonella species in acute diarrhoea in Hamedan, Islamic Republic of Iran. 2007. 17684844

[14] Aslani MM, Badami N, Mahmoodi M, Bouzari S. Verotoxin-producing Escherichia coli (VTEC) infection in randomly selected population of Ilam Province (Iran). Scandinavian journal of infectious diseases. 1998;30(5):473–6. 10.1080/00365549850161467 10066047

[15] Aslani MM, Bouzari S. An epidemiological study on Verotoxin-producing Escherichia coli (VTEC) infection among population of northern region of Iran (Mazandaran and Golestan provinces). European journal of epidemiology. 2003;18(4):345–9. 10.1023/a:1023602416726 12803375

[16] Willshaw GA, Cheasty T, Smith HR, O’Brien SJ, Adak GK. Verotoxin-producing Escherichia coli (VTEC) O157 and other VTEC from human infection in England and Wales: 1995–1998. J Med Microbiol 2001;50(2):135–42. 10.1099/0022-1317-50-2-135 11211220

[17] Payne SM, Finkelstein RA. Detection and differentiation of iron-responsive avirulent mutants on Congo red agar. Infection and immunity. 1977;18(1):94–8. 10.1128/IAI.18.1.94-98.1977 409688 PMC421198

[18] Eybpoosh S, Fazlalipour M, Baniasadi V, Pouriayevali MH, Sadeghi F, Ahmadi Vasmehjani A, et al Epidemiology of West Nile Virus in the Eastern Mediterranean region: A systematic review. PLoS neglected tropical diseases. 2019;13(1):e0007081 10.1371/journal.pntd.0007081 30695031 PMC6368338

[19] Caprioli A, Morabito S, Brugère H, Oswald E. Enterohaemorrhagic Escherichia coli: emerging issues on virulence and modes of transmission. Veterinary research. 2005;36(3):289–311. 10.1051/vetres:2005002 15845227

[20] Olsen SJ, Miller G, Breuer T, Kennedy M, Higgins C, Walford J, et al A waterborne outbreak of Escherichia coli O157: H7 infections and hemolytic uremic syndrome: implications for rural water systems. Emerging infectious diseases. 2002;8(4):370 11971769 10.3201/eid0804.000218 PMC2730238

[21] Ihekweazu C, Carroll K, Adak B, Smith G, Pritchard G, Gillespie I, et al Large outbreak of verocytotoxin-producing Escherichia coli O157 infection in visitors to a petting farm in South East England, 2009. Epidemiology & Infection. 2012;140(8):1400–13. 22093751 10.1017/S0950268811002111 PMC3404481

[22] Gonzalez-Escalona N, Kase JA. Virulence gene profiles and phylogeny of Shiga toxin-positive Escherichia coli strains isolated from FDA regulated foods during 2010–2017. Plos one. 2019;14(4):e0214620 10.1371/journal.pone.0214620 30934002 PMC6443163

[23] Jafari F, Shokrzadeh L, Hamidian M, Salmanzadeh-Ahrabi S, Zali MR. Acute diarrhea due to enteropathogenic bacteria in patients at hospitals in Tehran. Jpn J Infect Dis. 2008;61(4):269–73. 18653967

[24] Jafari F, Garcia-Gil L, Salmanzadeh-Ahrabi S, Shokrzadeh L, Aslani M, Pourhoseingholi M, et al Diagnosis and prevalence of enteropathogenic bacteria in children less than 5 years of age with acute diarrhea in Tehran children’s hospitals. Journal of infection. 2009;58(1):21–7. 10.1016/j.jinf.2008.10.013 19117609

[25] Jafari F, Hamidian M, Rezadehbashi M, Doyle M, Salmanzadeh-ahrabi S, Derakhshan F, et al Prevalence and antimicrobial resistance of diarrheagenic Escherichia coli and Shigella species associated with acute diarrhea in Tehran, Iran. Canadian journal of infectious diseases and medical microbiology. 2009;20(3):e56–e62. 10.1155/2009/341275 20808457 PMC2770303

[26] Gupta S, Keck J, Ram P, Crump J, Miller M, Mintz E. Part III. Analysis of data gaps pertaining to enterotoxigenic Escherichia coli infections in low and medium human development index countries, 1984–2005. Epidemiology & Infection. 2008;136(6):721–38. 10.1017/S095026880700934X 17686197 PMC2870873

[27] Qadri F, Svennerholm A-M, Faruque A, Sack RB. Enterotoxigenic Escherichia coli in developing countries: epidemiology, microbiology, clinical features, treatment, and prevention. Clinical microbiology reviews. 2005;18(3):465–83. 10.1128/CMR.18.3.465-483.2005 16020685 PMC1195967

[28] Zhang H, Pan F, Zhao X, Wang G, Tu Y, Fu S, et al Distribution and antimicrobial resistance of enteric pathogens in Chinese paediatric diarrhoea: a multicentre retrospective study, 2008–2013. Epidemiology & Infection. 2015;143(12):2512–9. 10.1017/S0950268814003756 25586929 PMC9151064

[29] Yu J, Jing H, Lai S, Xu W, Li M, Wu J, et al Etiology of diarrhea among children under the age five in China: results from a five-year surveillance. Journal of Infection. 2015;71(1):19–27. 10.1016/j.jinf.2015.03.001 25753104 PMC4667737

[30] Al-Gallas N, Bahri O, Bouratbeen A, Haasen AB, Aissa RB. Etiology of acute diarrhea in children and adults in Tunis, Tunisia, with emphasis on diarrheagenic Escherichia coli: prevalence, phenotyping, and molecular epidemiology. The American journal of tropical medicine and hygiene. 2007;77(3):571–82. 17827382

[31] Vasconcelos MJdOB, Rissin A, Figueiroa JN, Lira PICd, Batista Filho M. Factors associated with diarrhea in children under five years old in the state of Pernambuco, according to surveys conducted in 1997 and 2006. Revista de saude publica. 2018;52:48 10.11606/s1518-8787.2018052016094 29723386 PMC5947442

[32] Karambu S, Matiru V, Kiptoo M, Oundo J. Characterization and factors associated with diarrhoeal diseases caused by enteric bacterial pathogens among children aged five years and below attending Igembe District Hospital, Kenya. Pan African Medical Journal. 2014;16(1). 10.11604/pamj.2013.16.37.2947 PMC3932116 24570797

[33] von Mentzer A, Connor TR, Wieler LH, Semmler T, Iguchi A, Thomson NR, et al Identification of enterotoxigenic Escherichia coli (ETEC) clades with long-term global distribution. Nature genetics. 2014;46(12):1321 10.1038/ng.3145 25383970

[34] Anderson JD IV, Bagamian KH, Muhib F, Amaya MP, Laytner LA, Wierzba T, et al Burden of enterotoxigenic Escherichia coli and shigella non-fatal diarrhoeal infections in 79 low-income and lower middle-income countries: a modelling analysis. The Lancet Global Health. 2019;7(3):e321–e30. 10.1016/S2214-109X(18)30483-2 30784633 PMC6379821

[35] Alizade H, Teshnizi SH, Azad M, Shojae S, Gouklani H, Davoodian P, et al An overview of diarrheagenic Escherichia coli in Iran: A systematic review and meta-analysis. Journal of Research in Medical Sciences: The Official Journal of Isfahan University of Medical Sciences. 2019;24 31007693 10.4103/jrms.JRMS_256_18 PMC6450139

[36] Nazarian S, Gargari SLM, Rasooli I, Alerasol M, Bagheri S, Alipoor SD. Prevalent phenotypic and genotypic profile of enterotoxigenic Escherichia coli among Iranian children. Japanese Journal of Infectious Diseases. 2014;67(2):78–85. 10.7883/yoken.67.78 24647248

[37] Zeighami H, Haghi F, Hajiahmadi F, Kashefiyeh M, Memariani M. Multi-drug-resistant enterotoxigenic and enterohemorrhagic Escherichia coli isolated from children with diarrhea. Journal of Chemotherapy. 2015;27(3):152–5. 10.1179/1973947813Y.0000000161 24571245

[38] Haghi F, Zeighami H, Hajiahmadi F, Khoshvaght H, Bayat M. Frequency and antimicrobial resistance of diarrhoeagenic Escherichia coli from young children in Iran. Journal of medical microbiology. 2014;63(3):427–32. 10.1099/jmm.0.064600-0 24281909

[39] Kashefiyeh M, Zeighami H, Haghi F. Frequency of enterotoxigenic Escherichia coli isolates harboring eltb and elta genes in diarrheal specimens among children younger than 5 years in Tabriz hospitals. Journal of Zanjan University of Medical Sciences and Health Services. 2013;21(88).

[40] Wennerås C, Erling V. Prevalence of enterotoxigenic Escherichia coli-associated diarrhoea and carrier state in the developing world. Journal of Health, Population and Nutrition. 2004:370–82. 15663170

[41] Ochoa TJ, Barletta F, Contreras C, Mercado E. New insights into the epidemiology of enteropathogenic Escherichia coli infection. Transactions of the Royal Society of Tropical Medicine and Hygiene. 2008;102(9):852–6. 10.1016/j.trstmh.2008.03.017 18455741 PMC2575077

[42] SOLTAN DMM. Diarrhea caused by enteropathogenic bacteria in children. 2001.

[43] Dallal MS, Khorramizadeh M, MoezArdalan K. Occurrence of enteropathogenic bacteria in children under 5 years with diarrhoea in south Tehran. 2006. 17333824

[44] Bouzari S, Jafari A, Zarepoor M. Distribution of genes encoding toxins and antibiotic resistance patterns in diarrhoeagenic Escherichia coli isolates in Tehran. EMHJ-Eastern Mediterranean Health Journal, 13 (2), 287–293, 2007 2007. 17684850

[45] Shahbazi G, Hasani A, Asgharzadeh M, Rahim-Rahimi AA, Alebouyeh M. Prevalence of enteroaggregative Escherichia coli among children with gastroenteritis in the Northwest of Iran. Journal of Research in Clinical Medicine. 2015;3(3):183–9.

[46] Miri ST, Dashti A, Mostaan S, Kazemi F, Bouzari S. Identification of different Escherichia coli pathotypes in north and north-west provinces of Iran. Iranian journal of microbiology. 2017;9(1):33 28775821 PMC5534002

[47] Abbasi P, Kargar M, Doosti A, Mardaneh J, Ghorbani Dalini S, Dehyadegari MA. Real time pcr for characterization of enteroinvasive e. Coli (eiec) in children with diarrhea in shiraz. Annals of Colorectal Research. 2014;2(3):0-.

[48] Kalantar E, Solatni J, Khosravi B, Salehi A. Frequency of E. coli pathotypes in acute diarrhea of children and its related factorsat Beassat hospital, Sanandaj. Asian Pacific Journal of Tropical Medicine. 2009;2(4):64–6.

[49] Cohen MB, Nataro JP, Bernstein DI, Hawkins J, Roberts N, Staat MA. Prevalence of diarrheagenic Escherichia coli in acute childhood enteritis: a prospective controlled study. The Journal of pediatrics. 2005;146(1):54–61. 10.1016/j.jpeds.2004.08.059 15644823

[50] Shah M, Kathiiko C, Wada A, Odoyo E, Bundi M, Miringu G, et al Prevalence, seasonal variation, and antibiotic resistance pattern of enteric bacterial pathogens among hospitalized diarrheic children in suburban regions of central Kenya. Tropical medicine and health. 2016;44(1):39. 27942243 10.1186/s41182-016-0038-1 PMC5126808

[51] Huang W-C, Hsu B-M, Kao P-M, Tao C-W, Ho Y-N, Kuo C-W, et al Seasonal distribution and prevalence of diarrheagenic Escherichia coli in different aquatic environments in Taiwan. Ecotoxicology and environmental safety. 2016;124:37–41. 10.1016/j.ecoenv.2015.09.040 26454073

[52] Philipsborn R, Ahmed SM, Brosi BJ, Levy K. Climatic drivers of diarrheagenic Escherichia coli incidence: a systematic review and meta-analysis. The Journal of infectious diseases. 2016;214(1):6–15. 10.1093/infdis/jiw081 26931446 PMC4907410

