# Supplementary material for: Frequency of five Escherichia Coli pathotypes in Iranian adults and children with acute diarrhea
Source: PLoS One. 2021 Feb 4;16(2):e0245470. doi: 10.1371/journal.pone.0245470 (PMC7861387; doi:10.1371/journal.pone.0245470)
Supplement: S1 File — (DOCX) [file pone.0245470.s001.docx]

**S1 File. Frequency of *E. coli* pathotypes in 15 selected provinces of Iran**

**S1 - Table S1.** Frequency of five *E. coli* pathotypes in 15 selected provinces of Iran

|  | **STEC** | **ETEC** | **EPEC** | **EAEC** | **EIEC** | **Total pathotypes** | **Total received samples** | **Frequency*** | **95% CI** |
| --- | --- | --- | --- | --- | --- | --- | --- | --- | --- |
| **Provinces** | n | n | n | n | n | n | n | % | % |
| **East Azerbaijan** | 3 | 5 | 5 | 1 | 0 | 14 | 26 | 53.8 | 35.5, 71.2 |
| **Gilan** | 29 | 5 | 3 | 4 | 0 | 41 | 74 | 55.4 | 44.1, 66.2 |
| **Golestan** | 7 | 1 | 1 | 0 | 0 | 9 | 11 | 81.8 | 52.3, 94.9 |
| **Khorasan** | 3 | 11 | 2 | 0 | 0 | 16 | 46 | 34.8 | 22.7, 49.2 |
| **Sistan & Baluchestan** | 5 | 4 | 1 | 2 | 0 | 12 | 18 | 66.7 | 43.7, 83.7 |
| **Semnan** | 10 | 11 | 6 | 2 | 1 | 30 | 48 | 62.5 | 48.4, 74.8 |
| **Esfahan** | 3 | 17 | 0 | 3 | 0 | 23 | 54 | 42.6 | 30.3, 55.8 |
| **Kerman** | 59 | 15 | 12 | 1 | 1 | 88 | 108 | 81.5 | 73.1, 87.7 |
| **Hormozgan** | 36 | 3 | 7 | 0 | 0 | 46 | 60 | 76.7 | 64.6, 85.6 |
| **Khuzestan** | 49 | 21 | 51 | 5 | 1 | 127 | 164 | 77.4 | 70.5, 83.2 |
| **Kordestan** | 20 | 12 | 8 | 16 | 0 | 56 | 99 | 56.6 | 46.7, 65.9 |
| **Tehran** | 37 | 10 | 8 | 5 | 0 | 60 | 79 | 75.9 | 65.5, 84.0 |
| **Hamedan** | 13 | 16 | 11 | 4 | 0 | 44 | 83 | 53.0 | 42.4, 63.4 |
| **Zanjan** | 28 | 5 | 5 | 0 | 0 | 38 | 47 | 80.9 | 67.5, 89.6 |
| **Fars** | 45 | 1 | 9 | 0 | 0 | 55 | 62 | 88.7 | 78.5, 94.4 |
| **Total** | 347 | 137 | 129 | 43 | 3 | 659 | 979 | 67.3 | 64.3, 70.2 |

* Frequency is calculated by dividing total number of *E. coli* pathotypes identified in each province to the total number of *E. coli* samples received from that province.

**S1 - Table S2.** Frequency of the STEC pathotype in 15 selected provinces of Iran

| **Provinces** | **STEC** | **Received *E.coli* samples** | **Frequency*** |
| --- | --- | --- | --- |
|  | n | n | % (95% CI) |
| **East Azerbaijan** | 3 | 26 | 11.5 (2.4, 3.0) |
| **Gilan** | 29 | 74 | 39.1 (28.0, 51.2) |
| **Golestan** | 7 | 11 | 63.6 (30.7, 89.0) |
| **Khorasan** | 3 | 46 | 6.5 (1.3, 17.8) |
| **Sistan & Baluchestan** | 5 | 18 | 27.7 (9.6, 53.4) |
| **Semnan** | 10 | 48 | 20.8 (10.4, 34.9) |
| **Esfahan** | 3 | 54 | 5.5 (1.1, 15.3) |
| **Kerman** | 59 | 108 | 54.6 (44.7, 64.2) |
| **Hormozgan** | 36 | 60 | 60.0 (46.5, 72.4) |
| **Khuzestan** | 49 | 164 | 29.8 (22.9, 37.5) |
| **Kordestan** | 20 | 99 | 20.2 (12.7, 29.4) |
| **Tehran** | 37 | 79 | 46.8 (35.5, 58.4) |
| **Hamedan** | 13 | 83 | 15.6 (8.6, 25.2) |
| **Zanjan** | 28 | 47 | 59.5 (44.2, 37.6) |
| **Fars** | 45 | 62 | 72.5 (59.7, 83.1) |
| **Total** | 347 | 979 | 35.4 (32.4, 38.5) |

* Frequency is calculated by dividing total number of STEC pathotypes identified in each province to the total number of *E. coli* samples received from that province.

**S1 - Table S3.** Frequency of ETEC pathotype in 15 selected provinces of Iran

| **Provinces** | **ETEC** | **Received *E.coli* samples** | **Frequency*** |
| --- | --- | --- | --- |
|  | n | n | % (95% CI) |
| **East Azerbaijan** | 5 | 26 | 19.2 (6.5, 39.3) |
| **Gilan** | 5 | 74 | 6.7 (2.2, 15.0) |
| **Golestan** | 1 | 11 | 9.0 (0.2, 41.2) |
| **Khorasan** | 11 | 46 | 23.9 (22.5, 38.7) |
| **Sistan & Baluchestan** | 4 | 18 | 22.2 (6.4, 47.6) |
| **Semnan** | 11 | 48 | 22.9 (12.0, 37.3) |
| **Esfahan** | 17 | 54 | 31.4 (19.5, 45.5) |
| **Kerman** | 15 | 108 | 13.8 (7.9, 21.8) |
| **Hormozgan** | 3 | 60 | 5.0 (1.0, 31.9) |
| **Khuzestan** | 21 | 164 | 12.8 (8.1, 18.9) |
| **Kordestan** | 12 | 99 | 12.1 (6.4, 20.2) |
| **Tehran** | 10 | 79 | 12.6 (6.2, 22.0) |
| **Hamedan** | 16 | 83 | 19.2 (11.4, 29.4) |
| **Zanjan** | 5 | 47 | 10.6 (3.5, 23.1) |
| **Fars** | 1 | 62 | 1.6 (0.0, 8.6) |
| **Total** | 137 | 979 | 14.0(11.9, 16.3) |

* Frequency is calculated by dividing total number of ETEC pathotypes identified in each province to the total number of *E. coli* samples received from that province.

**S1 - Table S4.** Frequency of EPEC pathotype in 15 selected provinces of Iran

| **Provinces** | **EPEC** | **Received *E.coli* samples** | **Frequency*** |
| --- | --- | --- | --- |
|  | n | n | % (95% CI) |
| **East Azerbaijan** | 5 | 26 | 19.2 (6.5, 39.3) |
| **Gilan** | 3 | 74 | 4.0 (0.8, 11.3) |
| **Golestan** | 1 | 11 | 9.0 (0.2, 41.2) |
| **Khorasan** | 2 | 46 | 4.3 (0.5, 14.8) |
| **Sistan & Baluchestan** | 1 | 18 | 5.5 (0.1, 27.2) |
| **Semnan** | 6 | 48 | 1.2 (4.7, 25.2) |
| **Esfahan** | 0 | 54 | 0 |
| **Kerman** | 12 | 108 | 11.1 (5.8, 18.6) |
| **Hormozgan** | 7 | 60 | 11.7 (4.8, 22.6) |
| **Khuzestan** | 51 | 164 | 31.0 (24.1, 38.7) |
| **Kordestan** | 8 | 99 | 8.0 (3.5, 15.3) |
| **Tehran** | 8 | 79 | 10.1 (4.4, 18.9) |
| **Hamedan** | 11 | 83 | 13.2 (6.8, 22.4) |
| **Zanjan** | 5 | 47 | 10.6 (3.5, 23.1) |
| **Fars** | 9 | 62 | 14.5 (6.8, 25.7) |
| **Total** | 129 | 979 | 13.1 (11.1, 15.4) |

* Frequency is calculated by dividing total number of EPEC pathotypes identified in each province to the total number of *E. coli* samples received from that province.

**S1 - Table S5.** Frequency of EAEC pathotype in 15 selected provinces of Iran

|  | **EAEC** | **Received *E.coli* samples** | **Frequency*** |
| --- | --- | --- | --- |
| **Provinces** | n | n | % (95% CI) |
| **East Azerbaijan** | 1 | 26 | 3.8 (0.0, 19.6) |
| **Gilan** | 4 | 74 | 4.5 (1.4, 13.2) |
| **Golestan** | 0 | 11 | 0 |
| **Khorasan** | 0 | 46 | 0 |
| **Sistan & Baluchestan** | 2 | 18 | 11.1 (1.3, 34.7) |
| **Semnan** | 2 | 48 | 4.1 (0.5, 14.2) |
| **Esfahan** | 3 | 54 | 5.5 (1.1, 15.3) |
| **Kerman** | 1 | 108 | 0.9 (0.0, 5.0) |
| **Hormozgan** | 0 | 60 | 0 |
| **Khuzestan** | 5 | 164 | 3.0 (0.9, 6.9) |
| **Kordestan** | 16 | 99 | 6.1 (9.5, 24.9) |
| **Tehran** | 5 | 79 | 6.3 (2.0, 14.1) |
| **Hamedan** | 4 | 83 | 4.8 (1.3, 11.8) |
| **Zanjan** | 0 | 47 | 0 |
| **Fars** | 0 | 62 | 0 |
| **Total** | 43 | 979 | 4.3(3.1, 5.8) |

* Frequency is calculated by dividing total number of EAEC pathotypes identified in each province to the total number of *E. coli* samples received from that province.

**S1 - Table S6.** Frequency of EIEC pathotype in 15 selected provinces of Iran

| **Provinces** | **EIEC** | **Received *E.coli* samples** | **Frequency*** |
| --- | --- | --- | --- |
|  | n | n | % (95% CI) |
| **East Azerbaijan** | 0 | 26 | 0 |
| **Gilan** | 0 | 74 | 0 |
| **Golestan** | 0 | 11 | 0 |
| **Khorasan** | 0 | 46 | 0 |
| **Sistan & Baluchestan** | 0 | 18 | 0 |
| **Semnan** | 1 | 48 | 0.2 (0.0, 11.0) |
| **Esfahan** | 0 | 54 | 0 |
| **Kerman** | 1 | 108 | 0.9 (0.0, 0.5) |
| **Hormozgan** | 0 | 60 | 0 |
| **Khuzestan** | 1 | 164 | 0.6 (0.0, 3.3) |
| **Kordestan** | 0 | 99 | 0 |
| **Tehran** | 0 | 79 | 0 |
| **Hamedan** | 0 | 83 | 0 |
| **Zanjan** | 0 | 47 | 0 |
| **Fars** | 0 | 62 | 0 |
| **Total** | 3 | 979 | 0.3 (0.1, 0.9) |

* Frequency is calculated by dividing total number of EIEC pathotypes identified in each province to the total number of *E. coli* samples received from that province.
