# Supplementary material for: Frequency of five Escherichia Coli pathotypes in Iranian adults and children with acute diarrhea
Source: PLoS One. 2021 Feb 4;16(2):e0245470. doi: 10.1371/journal.pone.0245470 (PMC7861387; doi:10.1371/journal.pone.0245470)
Supplement: S2 File — (DOCX) [file pone.0245470.s002.docx]

**S2 File. Frequency of *E. coli* pathotypes in 43 selected cities of Iran**

|  | **STEC** | **EPEC** | **ETEC** | **EIEC** | **EAEC** | **Total pathogenic *E. coli* samples** | **Total *E. coli* samples received** | **Frequency*** |
| --- | --- | --- | --- | --- | --- | --- | --- | --- |
| **Cities** | n | n | n | n | n | N | N | % |
| **East Azerbaijan** |  |  |  |  |  |  |  |  |
| Tabriz | 0 | 0 | 1 | 0 | 0 | 1 | 9 | 11.1 |
| Sarab | 0 | 0 | 4 | 0 | 0 | 4 | 5 | 80.0 |
| Hashtrood | 3 | 5 | 0 | 0 | 1 | 9 | 12 | 75.0 |
| **Gilan** |  |  |  |  |  |  |  |  |
| Rasht | 12 | 2 | 3 | 0 | 1 | 18 | 21 | 85.7 |
| Soome-Sara | 0 | 0 | 0 | 0 | 1 | 1 | 5 | 20.0 |
| Langrood | 17 | 1 | 2 | 0 | 1 | 22 | 48 | 45.8 |
| **Golestan** |  |  |  |  |  |  |  |  |
| Gorgan | 3 | 0 | 0 | 0 | 0 | 3 | 3 | 100.0 |
| Minoodasht | 4 | 1 | 1 | 0 | 0 | 6 | 8 | 75.0 |
| **Razavi Khorasan** |  |  |  |  |  |  |  |  |
| Mashhad | 2 | 2 | 8 | 0 | 0 | 12 | 30 | 40.0 |
| Kashmar | 1 | 0 | 3 | 0 | 0 | 4 | 16 | 25.0 |
| **Sistan & Baluchistan** |  |  |  |  |  |  |  |  |
| Zahedan | 3 | 0 | 4 | 0 | 1 | 8 | 10 | 80.0 |
| Chabahar | 2 | 1 | 0 | 0 | 1 | 4 | 6 | 66.7 |
| Saravan | 0 | 0 | 0 | 0 | 0 | 0 | 2 | 0.0 |
| **Semnan** |  |  |  |  |  |  |  |  |
| Semnan | 10 | 6 | 10 | 1 | 1 | 28 | 43 | 65.1 |
| Damghan | 0 | 0 | 0 | 0 | 0 | 0 | 1 | 0.0 |
| Garmsar | 0 | 0 | 1 | 0 | 1 | 2 | 4 | 50.0 |
| **Esfahan** |  |  |  |  |  |  |  |  |
| Esfahan | 3 | 0 | 14 | 0 | 2 | 19 | 43 | 44.2 |
| Najaf Abad | 0 | 0 | 0 | 0 | 0 | 0 | 5 | 0.0 |
| Ardestan | 0 | 0 | 3 | 0 | 1 | 4 | 6 | 66.7 |
| **Kerman** |  |  |  |  |  |  |  |  |
| Kerman | 3 | 1 | 5 | 0 | 0 | 9 | 19 | 47.4 |
| Sirjan | 45 | 9 | 6 | 1 | 0 | 61 | 64 | 95.3 |
| Bafgh | 11 | 2 | 4 | 0 | 1 | 18 | 25 | 72.0 |
| **Hormozgan** |  |  |  |  |  |  |  |  |
| Bandar Abbas | 6 | 3 | 2 | 0 | 0 | 11 | 15 | 73.3 |
| Bandar Lengeh | 15 | 1 | 1 | 0 | 0 | 17 | 24 | 70.8 |
| Minab | 15 | 3 | 0 | 0 | 0 | 18 | 21 | 85.7 |
| **Khuzestan** |  |  |  |  |  |  |  |  |
| Ahvaz | 7 | 20 | 17 | 0 | 0 | 44 | 55 | 80.0 |
| Andimeshk | 21 | 22 | 4 | 1 | 4 | 52 | 74 | 70.3 |
| Shooshtar | 21 | 9 | 0 | 0 | 1 | 31 | 35 | 88.6 |
| **Kordestan** |  |  |  |  |  |  |  |  |
| Sanandaj | 0 | 1 | 0 | 0 | 0 | 1 | 3 | 33.3 |
| Saghez | 12 | 4 | 9 | 0 | 10 | 35 | 54 | 64.8 |
| Marivan | 8 | 3 | 3 | 0 | 6 | 20 | 42 | 47.6 |
| **Tehran** |  |  |  |  |  |  |  |  |
| Tehran | 28 | 7 | 9 | 0 | 5 | 49 | 60 | 81.7 |
| Rey | 2 | 1 | 1 | 0 | 0 | 3 | 10 | 30.0 |
| Eslamshahr | 7 | 0 | 0 | 0 | 0 | 8 | 9 | 88.9 |
| **Hamedan** |  |  |  |  |  |  |  |  |
| Hamedan | 2 | 3 | 6 | 0 | 1 | 12 | 14 | 85.7 |
| Kaboodar Ahang | 3 | 2 | 2 | 0 | 1 | 8 | 21 | 38.1 |
| Nahavand | 8 | 6 | 8 | 0 | 2 | 24 | 48 | 50.0 |
| **Zanjan** |  |  |  |  |  |  |  |  |
| Zanjan | 0 | 0 | 1 | 0 | 0 | 1 | 3 | 33.3 |
| Abhar | 25 | 5 | 4 | 0 | 0 | 34 | 41 | 82.9 |
| Tarem | 3 | 0 | 0 | 0 | 0 | 3 | 3 | 100.0 |
| **Fars** |  |  |  |  |  |  |  |  |
| Shiraz | 25 | 6 | 1 | 0 | 0 | 32 | 35 | 91.4 |
| Abadeh | 7 | 2 | 0 | 0 | 0 | 9 | 9 | 100.0 |
| Lar | 13 | 1 | 0 | 0 | 0 | 14 | 18 | 77.8 |
| **Total** | **347** | **129** | **137** | **3** | **43** | **659** | **979** | **67.3** |

* Frequency is calculated by dividing total number of *E. coli* pathotypes identified in each city to the total number of *E. coli* samples received from that city.
