# Supplementary material for: Frequency of five Escherichia Coli pathotypes in Iranian adults and children with acute diarrhea
Source: PLoS One. 2021 Feb 4;16(2):e0245470. doi: 10.1371/journal.pone.0245470 (PMC7861387; doi:10.1371/journal.pone.0245470)
Supplement: S3 File — (DOCX) [file pone.0245470.s003.docx]

**S3 File. Seasonal trend of *E. coli* pathotypes in 15 selected provinces of Iran**

**S3 - Table S1. Seasonal trend of *E. coli* pathotypes in East Azerbaijan province**

| **Season** | **STEC** | **EPEC** | **ETEC** | **EIEC** | **EAEC** | **Total pathogenic samples** | **Total samples received** |
| --- | --- | --- | --- | --- | --- | --- | --- |
|  |  |  |  |  |  | n (%) |  |
| **Spring** | 0 (0) | 2 (0) | 2 (0) | 0 (0) | 0 (0) | 4 (57.1) | 7 (100) |
| **Summer** | 0 (0) | 1 (12.5) | 3 (37.5) | 0 (0) | 1 (12.5) | 5 (62.5) | 8 (100) |
| **Fall** | 3 (30.0) | 2 () | 0 (0) | 0 (0) | 0 (0) | 5 (50) | 10 (100) |
| **Winter** | 0 (0) | 0 (0) | 0 (0) | 0 (0) | 0 (0) | 0 (0) | 0 (100) |
| **Total** | 3 | 5 | 5 | 0 | 1 | 14 | 26 |

**S3 - Figure S1. Seasonal trend of *E. coli* pathotypes in East Azerbaijan province**

**S3 - Table S2. Seasonal trend of *E. coli* pathotypes in Gilan province**

| **Season** | **STEC** | **EPEC** | **ETEC** | **EIEC** | **EAEC** | **Total pathogenic samples (%)** | **Total samples received** |
| --- | --- | --- | --- | --- | --- | --- | --- |
| **Spring** | 1 (6.7) | 1 (6.7) | 3 (20.0) | 0 (0) | 0 (0) | 5 (33.3) | 15 (100) |
| **Summer** | 10 (41.7) | 0 (0) | 1 (4.2) | 0 (0) | 3 (12.5) | 14 (58.3) | 24 (100) |
| **Fall** | 10 (43.5) | 2 (8.7) | 0 (0) | 0 (0) | 1 (4.3) | 13 (56.5) | 23 (100) |
| **Winter** | 8 (66.7) | 0 (0) | 1 (8.3) | 0 (0) | 0 (0) | 9 (75.0) | 12 (100) |
| **Total** | 29 | 3 | 5 | 0 | 4 | 41 | 74 |

**S3 - Figure S2. Seasonal trend of *E. coli* pathotypes in Gilan province**

**S3 - Table S3. Seasonal trend of *E. coli* pathotypes in Golestan province**

| **Season** | **STEC** | **EPEC** | **ETEC** | **EIEC** | **EAEC** | **Total pathogenic samples (%)** | **Total samples received** |
| --- | --- | --- | --- | --- | --- | --- | --- |
| **Spring** | 0 | 0 | 0 | 0 | 0 | 0 | 0 |
| **Summer** | 0 | 0 | 0 | 0 | 0 | 0 | 0 |
| **Fall** | 4 | 1 | 1 | 0 | 0 | 6 | 8 |
| **Winter** | 3 | 0 | 0 | 0 | 0 | 3 | 3 |
| **Total** | 7 | 1 | 1 | 0 | 0 | 9 | 11 |

Due to scarcity of data, seasonal trend could not be inferred for Golestan province. Due to the same reason, we have not provided a graph for the seasonal trend. We also did not calculate relative frequency due to the same reason.

**S3 - Table S4. Seasonal trend of *E. coli* pathotypes in Razavi Khorasan province**

| **Season** | **STEC** | **EPEC** | **ETEC** | **EIEC** | **EAEC** | **Total pathogenic samples (%)** | **Total samples received** |
| --- | --- | --- | --- | --- | --- | --- | --- |
| **Spring** | 0 (0) | 0 (0) | 1 (33.3) | 0 (0) | 0 (0) | 1 (33.3) | 3 (100) |
| **Summer** | 1 (5.9) | 1 (5.9) | 5 (29.4) | 0 (0) | 0 (0) | 7 (41.2) | 17 (100) |
| **Fall** | 2 (18.2) | 1 (9.1) | 1 (9.1) | 0 (0) | 0 (0) | 4 (36.4) | 11 (100) |
| **Winter** | 0 (0) | 0 (0) | 4 (26.7) | 0 (0) | 0 (0) | 4 (26.7) | 15 (100) |
| **Total** | 3 | 2 | 11 | 0 | 0 | 16 | 46 |

Due to scarcity of data especially in spring and fall, interpretation of seasonal trend for this province should be made with caution.

**S3 - Figure S4. Seasonal trend of *E. coli* pathotypes in Razavi Khorasan province**

**S3 - Table S5. Seasonal trend of *E. coli* pathotypes in Sistan & Baluchestan province**

| **Season** | **STEC** | **EPEC** | **ETEC** | **EIEC** | **EAEC** | **Total pathogenic samples (%)** | **Total samples received** |
| --- | --- | --- | --- | --- | --- | --- | --- |
| **Spring** | 1 | 0 | 1 | 0 | 0 | 2 | 3 |
| **Summer** | 0 | 1 | 0 | 0 | 0 | 1 | 2 |
| **Fall** | 3 | 0 | 3 | 0 | 0 | 6 | 9 |
| **Winter** | 1 | 0 | 0 | 0 | 2 | 3 | 4 |
| **Total** | 5 | 1 | 4 | 0 | 2 | 12 | 18 |

Due to scarcity of data, seasonal trend could not be inferred for Sistan & Baluchestan province. Due to the same reason, we have not provided a graph for the seasonal trend. We also did not calculate relative frequency due to the same reason.

**S3 - Table S6. Seasonal trend of *E. coli* pathotypes in Semnan province**

| **Season** | **STEC** | **EPEC** | **ETEC** | **EIEC** | **EAEC** | **Total pathogenic samples (%)** | **Total samples received** |
| --- | --- | --- | --- | --- | --- | --- | --- |
| **Spring** | 0 (0) | 0 (0) | 2 (28.6) | 0 () | 0 (0) | 2 (28.6) | 7 (100) |
| **Summer** | 2 (13.3) | 2 (13.3) | 2 (13.3) | 1 (6.7) | 0 (0) | 7 (46.7) | 15 (100) |
| **Fall** | 3 (30.0) | 2 (20.0) | 4 (40.0) | 0 (0) | 1 (10.0) | 10 (100.0) | 10 (100) |
| **Winter** | 5 (31.3) | 2 (12.5) | 3 (18.8) | 0 (0) | 1 (6.3) | 11 (68.8) | 16 (100) |
| **Total** | 10 | 6 | 11 | 1 | 2 | 30 | 48 |

Due to scarcity of data especially in spring and fall, interpretation of seasonal trend for this province should be made with caution.

**S3 - Figure S6. Seasonal trend of E. coli pathotypes in Semnan province**

**S3 - Table S7. Seasonal trend of *E. coli* pathotypes in Esfahan province**

| **Season** | **STEC** | **EPEC** | **ETEC** | **EIEC** | **EAEC** | **Total pathogenic samples (%)** | **Total samples received** |
| --- | --- | --- | --- | --- | --- | --- | --- |
| **Spring** | 0 (0) | 0 (0) | 0 (0) | 0 (0) | 0 (0) | 0 (0) | 4 (100) |
| **Summer** | 0 (0) | 0 (0) | 7 (53.8) | 0 (0) | 2 (15.4) | 9 (69.2) | 13 (100) |
| **Fall** | 0 (0) | 0 (0) | 6 (22.2) | 0 (0) | 1 (3.7) | 7 (25.9) | 27 (100) |
| **Winter** | 3 (30.0) | 0 (0) | 4 (40.0) | 0 (0) | 0 (0) | 7 (70.0) | 10 (100) |
| **Total** | 3 | 0 | 17 | 0 | 3 | 23 | 54 |

Due to scarcity of data especially in spring and winter, interpretation of seasonal trend for this province should be made with caution.

**S3 - Figure S7. Seasonal trend of *E. coli* pathotypes in Esfahan province**

**S3 - Table S8. Seasonal trend of *E. coli* pathotypes in Kerman province**

| **Season** | **STEC** | **EPEC** | **ETEC** | **EIEC** | **EAEC** | **Total pathogenic samples (%)** | **Total samples received** |
| --- | --- | --- | --- | --- | --- | --- | --- |
| **Spring** | 11 (64.7) | 1 (5.9) | 2 (11.8) | 0 (0) | 0 0 (0) | 14 (82.4) | 17 (100) |
| **Summer** | 17 (68.0) | 0 (0) | 3 (12.0) | 0 (0) | 0 (0) | 20 (80.0) | 25 (100) |
| **Fall** | 20 (60.6) | 1 () | 5 (15.2) | 1 (3.0) | 1 (3.0) | 28 (84.8) | 33 (100) |
| **Winter** | 11 (33.3) | 10 (30.3) | 5 (15.2) | 0 (0) | 0 (0) | 26 (78.8) | 33 (100) |
| **Total** | 59 | 12 | 15 | 1 | 1 | 88 | 108 |

**S3 - Figure S8. Seasonal trend of *E. coli* pathotypes in Kerman province**

**S3 - Table S9. Seasonal trend of *E. coli* pathotypes in Hormozgan province**

| **Season** | **STEC** | **EPEC** | **ETEC** | **EIEC** | **EAEC** | **Total pathogenic samples (%)** | **Total samples received** |
| --- | --- | --- | --- | --- | --- | --- | --- |
| **Spring** | 4 (57.1) | 2 (28.6) | 0 (0) | 0 (0) | 0 (0) | 6 (85.7) | 7 (100) |
| **Summer** | 19 (70.4) | 2 (7.4) | 1 (3.7) | 0 (0) | 0 (0) | 22 (81.5) | 27 (100) |
| **Fall** | 10 (71.4) | 0 (0) | 1 (7.1) | 0 (0) | 0 (0) | 11 (78.6) | 14 (100) |
| **Winter** | 3 (25.0) | 3 (25.0) | 1 (8.3) | 0 (0) | 0 (0) | 7 (58.3) | 12 (100) |
| **Total** | 36 | 7 | 3 | 0 | 0 | 46 | 60 |

Due to scarcity of data in spring, interpretation of seasonal trend for this province should be made with caution.

**S3 - Figure S9. Seasonal trend of *E. coli* pathotypes in Hormozgan province**

**S3 - Table S10. Seasonal trend of *E. coli* pathotypes in Khuzestan province**

| **Season** | **STEC** | **EPEC** | **ETEC** | **EIEC** | **EAEC** | **Total pathogenic samples (%)** | **Total samples received** |
| --- | --- | --- | --- | --- | --- | --- | --- |
| **Spring** | 10 (31.3) | 15 (46.9) | 4 (12.5) | 0 (0) | 1 () | 30 (93.8) | 32 (100) |
| **Summer** | 10 (27.8) | 7 (19.4) | 7 (19.4) | 0 (0) | 1 () | 25 (69.4) | 36 (100) |
| **Fall** | 4 (12.1) | 10 (30.3) | 6 (18.2) | 1 (3.0) | 0 () | 21 (63.6) | 33 (100) |
| **Total** | 49 | 51 | 21 | 1 | 5 | 127 | 164 |

**S3 - Figure S10. Seasonal trend of *E. coli* pathotypes in Khuzestan province**

**S3 - Table S11. Seasonal trend of *E. coli* pathotypes in Kurdestan province**

| **Season** | **STEC** | **EPEC** | **ETEC** | **EIEC** | **EAEC** | **Total pathogenic samples (%)** | **Total samples received** |
| --- | --- | --- | --- | --- | --- | --- | --- |
| **Spring** | 0 (0) | 4 (23.5) | 7 (41.2) | 0 (0) | 2 ()11.8 | 13 (76.5) | 17 (100) |
| **Summer** | 5 (26.3) | 0 (0) | 2 (10.5) | 0 (0) | 1 (5.3) | 8 (42.1) | 19 (100) |
| **Fall** | 6 (22.2) | 2 (7.4) | 0 (0.0) | 0 (0) | 9 (33.3) | 17 (63.0) | 27 (100) |
| **Winter** | 9 (25.0) | 2 (5.6) | 3 (8.3) | 0 (0) | 4 (11.1) | 18 (50.0) | 36 (100) |
| **Total** | 20 | 8 | 12 | 0 | 16 | 56 | 99 |

**S3 - Figure S11. Seasonal trend of *E. coli* pathotypes in Kurdestan province**

**S3 - Table S12. Seasonal trend of *E. coli* pathotypes in Tehran province**

| **Season** | **STEC** | **EPEC** | **ETEC** | **EIEC** | **EAEC** | **Total pathogenic samples (%)** | **Total samples received** |
| --- | --- | --- | --- | --- | --- | --- | --- |
| **Spring** | 1 (7.7) | 1 (7.7) | 5 (38.5) | 0 (0) | 2 (15.4) | 9 (69.2) | 13 (100) |
| **Summer** | 8 (44.4) | 1 (5.6) | 1 (5.6) | 0 (0) | 3 (16.7) | 13 (72.2) | 18 (100) |
| **Fall** | 15 (75.0) | 2 (10.0) | 0 (0) | 0 (0) | 0 (0) | 17 (85.0) | 20 (100) |
| **Winter** | 13 (46.4) | 4 (14.3) | 4 (14.3) | 0 (0) | 0 (0) | 21 (75.0) | 28 (100) |
| **Total** | 37 | 8 | 10 | 0 | 5 | 60 | 79 |

**S3 - Figure S12. Seasonal trend of *E. coli* pathotypes in Tehran province**

**S3 - Table S13. Seasonal trend of *E. coli* pathotypes in Hamedan province**

| **Season** | **STEC** | **EPEC** | **ETEC** | **EIEC** | **EAEC** | **Total pathogenic samples (%)** | **Total samples received** |
| --- | --- | --- | --- | --- | --- | --- | --- |
| **Spring** | 3 (15.8) | 2 (10.5) | 8 (42.1) | 0 (0) | 2 (10.5) | 15 (78.9) | 19 (100) |
| **Summer** | 5 (25.0) | 3 (15.0) | 2 (10.0) | 0 (0) | 1 (5.0) | 11 (55.0) | 20 (100) |
| **Fall** | 4 (28.6) | 1 (7.1) | 1 (7.1) | 0 (0) | 0 (0) | 6 (42.9) | 14 (100) |
| **Winter** | 1 (3.3) | 5 (16.7) | 5 (16.7) | 0 (0) | 1 () | 12 (40.0) | 30 (100) |
| **Total** | 13 | 11 | 16 | 0 | 4 | 44 | 83 |

**S3 - Figure S13. Seasonal trend of *E. coli* pathotypes in Hamedan province**

**S3 - Table S14. Seasonal trend of *E. coli* pathotypes in Zanjan province**

| **Season** | **STEC** | **EPEC** | **ETEC** | **EIEC** | **EAEC** | **Total pathogenic samples (%)** | **Total samples received** |
| --- | --- | --- | --- | --- | --- | --- | --- |
| **Spring** | 3 (42.9) | 4 (57.1) | 0 (0) | 0 (0) | 0 (0) | 7 (100) | 7 (100) |
| **Summer** | 8 (50.0) | 1 (6.3) | 5 (31.3) | 0 (0) | 0 (0) | 14 (87.5) | 16 (100) |
| **Fall** | 11 (100.0) | 0 (0) | 0 (0) | 0 (0) | 0 (0) | 11 (100) | 11 (100) |
| **Winter** | 6 (46.2) | 0 (0) | 0 (0) | 0 (0) | 0 (0) | 6 (46.2) | 13 (100) |
| **Total** | 28 | 5 | 5 | 0 | 0 | 38 | 47 |

**S3 - Figure S14. Seasonal trend of *E. coli* pathotypes in Zanjan province**

**S3 - Table S15. Seasonal trend of *E. coli* pathotypes in Fars province**

| **Season** | **STEC** | **EPEC** | **ETEC** | **EIEC** | **EAEC** | **Total pathogenic samples (%)** | **Total samples received** |
| --- | --- | --- | --- | --- | --- | --- | --- |
| **Spring** | 10 (100) | 0 | 0 | 0 | 0 | 10 (100) | 10 (100) |
| **Summer** | 16 (80.0) | 1 (5.0) | 0 | 0 | 0 | 17 (85.0) | 20 (100) |
| **Fall** | 13 (100) | 0 | 0 | 0 | 0 | 13 (100) | 13 (100) |
| **Winter** | 6 (31.6) | 8 (42.1) | 1 (5.3) | 0 | 0 | 15 (78.9) | 19 (100) |
| **Total** | 45 | 9 | 1 | 0 | 0 | 55 | 62 |

**S3 - Figure S15. Seasonal trend of *E. coli* pathotypes in Fars province**
